# Epidural subtemporal intertentorial approach to the mesiotemporal lobe and lateral midbrain

**DOI:** 10.1016/j.bas.2025.105626

**Published:** 2025-10-06

**Authors:** David R. Peters, Lorenzo Giammattei, Pablo Gonzalez-Lopez, Giulia Cossu, Mercy George, Mahmoud Messerer, Daniele Starnoni, Roy T. Daniel

**Affiliations:** a Department of Neurosurgery, Lausanne University Hospital, Lausanne, Switzerland; b Faculty of Biology and Medicine, University of Lausanne, Lausanne, Switzerland; c Atrium Health, Department of Neurosurgery, Charlotte, NC, USA; d Carolina Neurosurgery & Spine Associates, Charlotte, NC, USA; e Department of Neurosurgery, Hospital General Universitario Alicante, Alicante, Spain; f Department of Otorhinolaryngology and Head and Neck Surgery, Lausanne University Hospital, Lausanne, Switzerland; g Lukas Lundin and Family Brain Tumor Research Center, Lausanne, Switzerland

**Keywords:** Epidural subtemporal intertentorial approach, Lateral midbrain, Mesiotemporal lobe, Subtemporal, Tentorial peeling

## Abstract

**Objective:**

The mesiotemporal lobe and lateral midbrain reside in a deep and complex anatomical region. The classic intradural subtemporal approach provides good access to this region but has significant risk of iatrogenic injury to the temporal lobe and vein of Labbé. We developed a new approach to this region to decrease the risk of iatrogenic injury.

**Methods:**

Anatomical dissection of three adult injected non-formalin fixed cadaveric heads was performed to evaluate the feasibility of performing an epidural subtemporal intertentorial approach (ESIA) to the mesiotemporal lobe and lateral midbrain. The surgical technique was analyzed in detail with accompanying cadaveric images and video used to describe the approach.

**Results:**

Tentorial peeling without petrosal drilling during the approach separated the tentorium into two layers, the posterior fossa tentorial leaf (PFTL) and the temporal tentorial leaf (TTL). Dural incision in the medial TTL allowed access to the mesiotemporal lobe, lateral midbrain, crural cistern, and ambient cistern. The lateral portion of the TTL and temporal dura remained intact, protecting the basal temporal lobe and vein of Labbé. Anatomic landmarks to determine the extent of tentorial peeling and site of the durotomy for access to the basal surface of the temporal lobe and lateral midbrain were identified.

**Conclusion:**

The ESIA is a novel approach for lesions of the mesiotemporal lobe and lateral midbrain. It provides direct access and a short working distance with reduced risk of iatrogenic injury to the temporal lobe and basal temporal veins.

## Highlights

• ESIA is a novel approach to access the mesial temporal lobe and mibrain.
• This epidural approach with tentorial peeling protects the temporal lobe and vein of Labbé.
• Cavernomas, gliomas, or other tumors may be directly accessed via the shortest possible working distance.
• More robust clinical experience is needed to understand advantages and disadvantages of ESIA.

## 1 Introduction

The mesiotemporal lobe (MTL) and lateral midbrain (LM) reside in a complex anatomical region containing many critical neurovascular structures. Many approaches have been described to access this area, each with its own set of advantages and disadvantages. The five main approaches to this region include transcortical, transsylvian, subtemporal, transorbital, and supracerebellar transtentorial. The primary drawback to most approaches is that they risk injury to adjacent normal structures either directly (i.e., transcortical approaches) or indirectly (i.e., retraction injury). The deep location of this region increases these risks.

Recently, a technique of tentorial peeling during a combined transpetrosal approach has been developed (Vidal et al., 2020; Giammattei et al., 2022, 2025), potentially reducing complications associated with the traditional combined petrosal approach by decreasing the risk of temporal lobe injury. We have applied the concept of tentorial peeling, without the need for petrosectomy, to create a new approach to the MTL and LM. The epidural subtemporal intertentorial approach (ESIA) provides direct and safe access to this region, minimizing disruption of normal brain. We aimed to explore our new approach though cadaveric dissections, with the objective of providing a detailed description of the technique, surgical anatomy, and identifying potential landmarks to develop a safe, reproducible, and efficient procedure.

## 2 Materials and methods

The surgical anatomy of the ESIA was described using three adult cadaver heads (2 male, 1 female; ages 72–83 years) injected with colored latex, non-formalin fixed. The procedure was performed with standard microsurgical instruments, high-speed drill (Midas Rex; Medtronic, Minneapolis, MN, USA), and a surgical microscope (Leica Microsystem, Wetzler, Germany). The dissection video was recorded using a 2D/4K camera (Karl Storz GmbH, KG, Tuttlingen, Germany) connected to the microscope. Each step of the surgical technique was described in detail with accompanying cadaveric pictures to describe the approach. A video illustrating the cadaveric dissection in detail was created (video 1). Local ethics committee approval was obtained for the use of cadavers and publication of the images. All potential patient identifiable information for the cadavers and the case example were removed. The patient in the case example consented to the procedure.

Supplementary data related to this article can be found online at https://doi.org/10.1016/j.bas.2025.105626

The following are the Supplementary data related to this article:

Video S1 Cadaveric dissection describing the epidural subtemporal intertentorial approach followed by an operative case. Video S1

## 3 Results

### 3.1 Head position and craniotomy

**Fig. 1:**
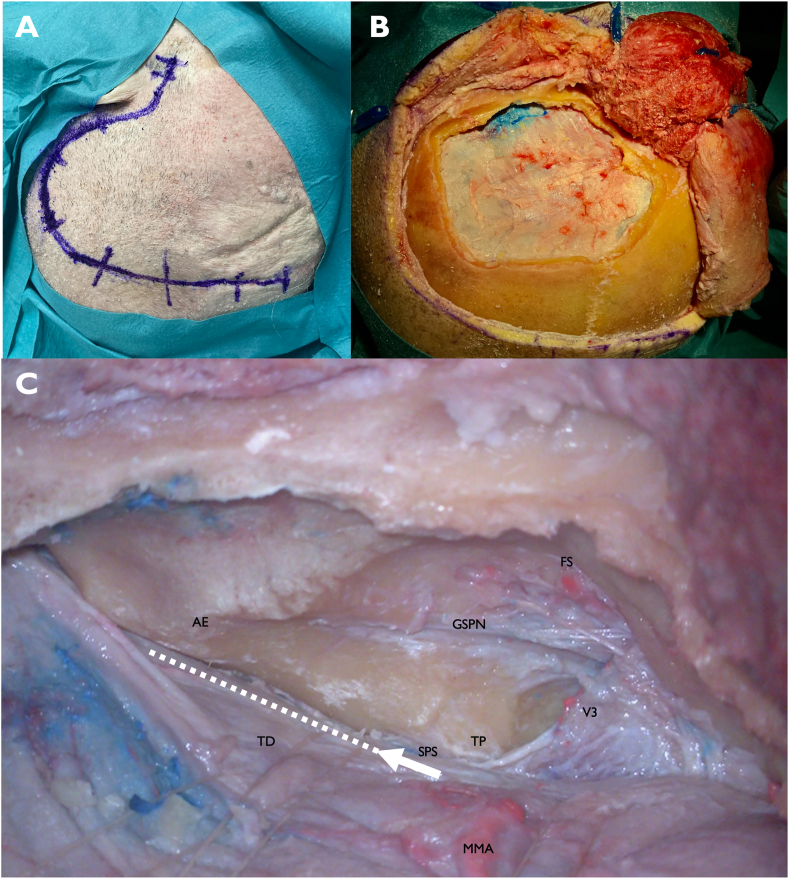
A) Question mark skin incision from the root of the zygoma extending superiorly above the pinna, posteriorly behind the ear, and curving anteriorly above the superior temporal line B) Wide craniotomy along the middle fossa floor is performed and the inferior edge of the craniotomy is drilled flat with the floor of the middle fossa. Wide dural elevation along the middle fossa floor in the anterior-posterior direction greatly improves visualization and reduces the force of epidural retraction on the temporal lobe C) Epidural dissection of the middle fossa floor is completed. The white arrow and dotted line represent the starting point for the incision in the temporal dura and the direction of the temporal peeling. The superior petrosal sinus (SPS) is easily identified near the posterior limit of V3. The proper plane for peeling is superior to the SPS and in between the posterior fossa tentorial leaf (PFTL) and temporal tentorial leaf (TTL) FS: foramen spinosum, GSPN: greater superficial petrosal nerve, AE: arcuate eminence, TP: trigeminal prominence, TD: temporal dura, SPS: superior petrosal sinus, V3: mandibular branch of the trigeminal nerve, MMA: middle meningeal artery (cut at the level of the foramen spinosum).

The specimens were fixed in a Mayfield head holder with 70–80° rotation and the vertex tilted slightly downward. A question mark incision was made (Fig. 1A). The skin flap and temporalis muscle were elevated in two layers, and an inter-fascial dissection was performed. At least 2 cm of temporal bone anterior to the zygomatic root was exposed. Burr holes were placed at the root of the zygoma, anterosuperior to the transverse sigmoid junction, and just below the superior temporal line. A wide middle fossa craniotomy was created, exposing the middle fossa floor and the temporal dura (Fig. 1B). The inferior edge of the craniotomy was drilled until it was flat with the floor of the middle fossa. Care was taken to ensure that the dura was not transgressed during the craniotomy.

### 3.2 Epidural dissection and tentorial peeling

Epidural dissection along the middle fossa floor was performed. The middle meningeal artery was followed down to the foramen spinosum, dissected free, and then coagulated and cut. Once cut, the dura was further mobilized in a posterior to anterior direction to avoid traction on the greater superficial petrosal nerve (GSPN) and geniculate ganglion (GG). The dura was cut and sharply dissected off V3 at the level of the foramen ovale to continue the epidural dissection. GSPN was sharply dissected free from the dura. Once V3 and GSPN were identified, the dura was further elevated posteriorly near to the sigmoid sinus and medially until the petrous ridge was reached. A wide dural elevation along the middle fossa floor provided better visualization and reduced the force of epidural retraction on the temporal lobe.

**Fig. 2:**
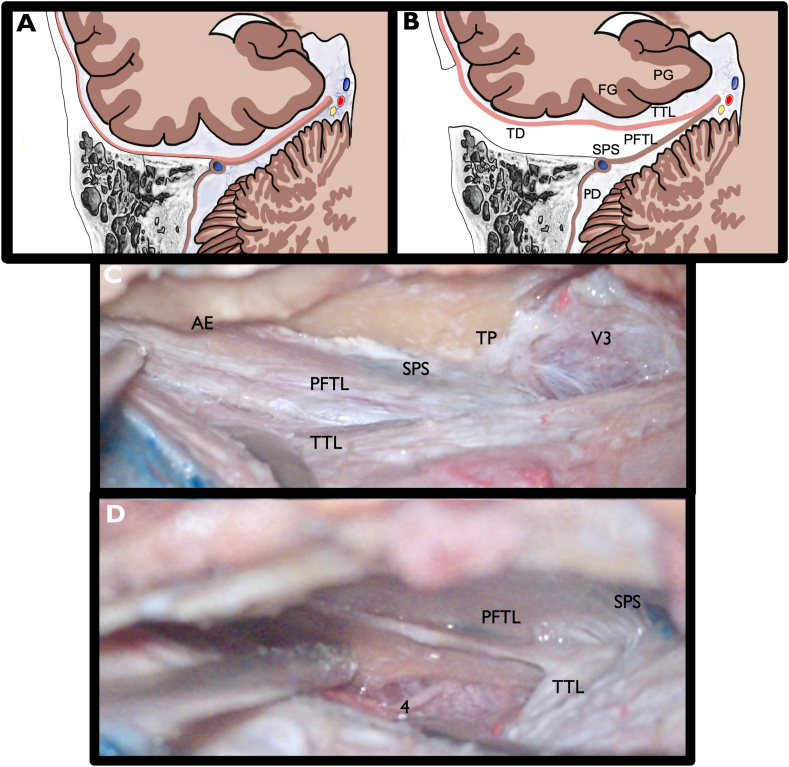
A–D) Illustration and cadaver images of tentorial peeling. A) Coronal view of the temporal lobe, tentorium, temporal bone, and cerebellum. The pink line indicates the temporal dura (TD), which is in continuity with the temporal tentorial leaf (TTL). The brown line indicates the presigmoid dura (PD), which is in continuity with the posterior fossa tentorial leaf (PFTL). B) The intertentorial space between the TTL and PFTL that is created by tentorial peeling, superior to the SPS. C) Cadaveric image after tentorial peeling is complete. SPS is unopened and stays attached to the petrous ridge. The intertentorial space has been developed, with clear separation of the PFTL and TTL. D) Dural incision has been made at the medial aspect of the TTL. The 4th cranial nerve and tentorial incisura are visible AE: arcuate eminence, TP: trigeminal prominence, SPS: superior petrosal sinus, V3: mandibular branch of the trigeminal nerve, TTL: temporal tentorial leaf, PFTL: posterior fossa tentorial leaf.

The tentorial peeling was started near the posterior limit of V3 and continued posteriorly in a direction parallel to the superior petrosal sinus (SPS) (Fig. 1C). The SPS was clearly identified prior to peeling. An incision was then made just superior to the SPS, and a plane was progressively developed in between the two layers of the tentorium, the temporal tentorial leaf (TTL) and the posterior fossa tentorial leaf (PFTL). The TTL is in anatomical continuity with the temporal dura (TD) of the middle fossa, and the PFTL is in anatomical continuity with the presigmoid dura (PD). Fig. 2A and B shows a schematic illustration of the approach and anatomy of the TD, TTL, PD, PFTL, and SPS. The SPS stayed connected to the PFTL and petrous ridge during the peeling. A wide dural peeling was performed, from just anterior to where the SPS enters the sigmoid sinus to V3 (Fig. 2C). The midpoint of the peeling in the anterior-posterior plane corresponded to the level of the quadrigeminal cistern.

### 3.3 Dural incision and temporal lobe anatomy

**Fig. 3:**
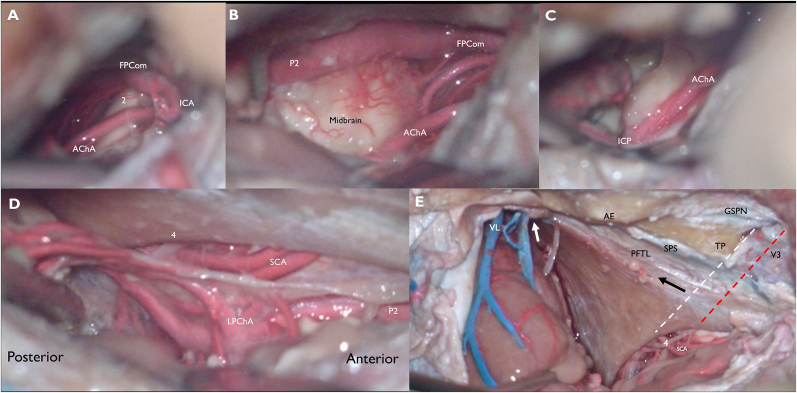
The parahippocampal gyrus (PG) and uncus have been subpially resected while the neurovascular structures have been preserved. This was done to better illustrate the relevant surrounding anatomy. A) Looking anteromedially, we see the dorsal internal carotid (ICA), the origin of a fetal posterior communicating artery (FPCom), the origin of the anterior choroidal artery (AChA), and the optic tract (2). B) The AChA and FPCom are followed distally, into the crural cistern. P2 segment of the posterior cerebral artery (PCA) is identified. The lateral midbrain is widely exposed C) The AChA is followed further distally until the inferior choroidal point (ICP) D) Looking posteromedially into the ambient cistern, a subpial view after resection of the PG reveals PCA branches including the lateral posterior choroidal arteries (LPChA). Infratentorially, the trochlear nerve (4) and duplicated superior cerebellar arteries (SCA) are visible. E) After completing the approach, the temporal dura and TTL was widely opened to better understand the relevant anatomy. White arrow highlights drainage of the vein of Labbé and basal temporal veins, undisturbed by the approach. Black arrow represents the level of the medial incision in the TTL that was used to reach the mesiotemporal lobe, just lateral to the medial extent of the peeling. The white dotted line is an imaginary line along the posterior edge of V3, and when it is continued medially, it approximates the posterior edge of the incisura. Red dotted line is an imaginary line that bisects and runs parallel to V3, and when it is continued medially to the incisura it approximates the location of the trochlear nerve entrance into the tentorium VL: vein of Labbé, AE: arcuate eminence, TP: trigeminal prominence, GSPN: greater superficial petrosal nerve, SPS: superior petrosal sinus, V3: mandibular branch of the trigeminal nerve, PFTL: posterior fossa tentorial leaf, 4: trochlear nerve, SCA: superior cerebellar artery.

Once the peeling had been extended far enough medially to reach the level of the parahippocampal gyrus (PG), an anterior-posterior incision in the TTL was made (Fig. 2D) approximately in the midpoint between the lateral convexity basal dura and the most medial extent of tentorial peeling. This provided a direct route to the MTL, LM, crural cistern (CC), and ambient cistern (AC) while keeping the rest of the basal and lateral temporal lobe along with the basal temporal veins covered by dura. This sagittal cut was placed at the level of the collateral sulcus, just lateral to the PG. An incision through the PG provides access to the temporal horn anteriorly and atrium posteriorly. A subpial resection of the PG and uncus was performed, and the neurovascular structures related to the MTL, LM, CC, and AC were identified (Fig. 3).

After completing the resection, the temporal dura and TTL were widely opened to better understand the relevant intradural anatomy. Basal temporal veins were undisturbed by the approach. The trochlear nerve was confirmed to be undisturbed by the approach. Two potentially useful landmarks were identified (Fig. 3). The first was an imaginary line that bisects and runs parallel to V3, continued medially to the incisura. This approximated the location of the trochlear nerve entrance into its groove below the tentorium. The second was an imaginary line parallel to the posterior edge of V3 continued to the incisura. This was a good estimate for the posterior margin of the incisura, where the tentorium extends across to the contralateral side behind the quadrigeminal cistern (Fig. 3E).

**Fig. 4:**
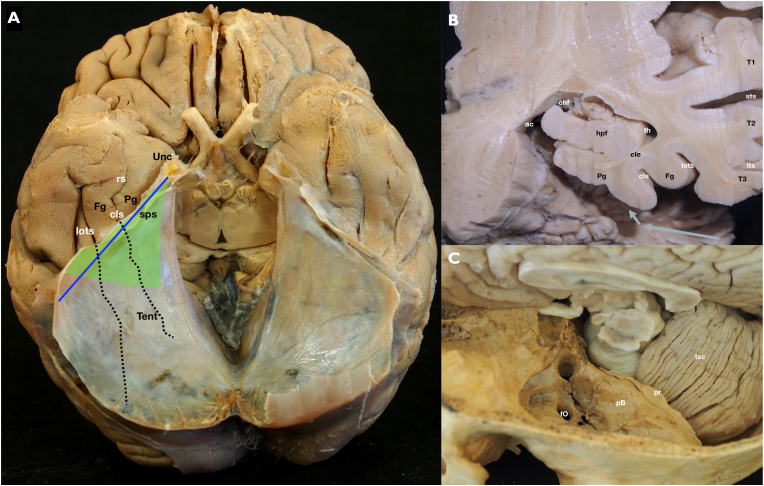
A–C) Anatomical specimens. A) Full brain specimen seen from below after a cut through the midbrain has been performed and the cerebellum and brainstem have been removed. The tentorium is shown covering the posterior halves of both temporal lobes. The collateral and lateral occipitotemporal sulci posterior extensions are hidden by the tentorium. Its relationship with the superior petrosal sinus (sps - blue line) is illustrated. The area of an effective tentorial peeling is seen (green area) to roughly expose the mesial structures of the temporal lobe. B) High magnification of a left temporal lobe coronal cut at the level of the midbrain tegmentum. The superior (T1), middle (T2) and inferior (T3) temporal gyri, as well as the superior (sts) and inferior (its) sulci are shown in the lateral aspect. The ventral stream and sagittal striatum fibers are longitudinally crossing the white matter located between the lateral cortex and the temporal horn (th). The close relationship between the collateral sulcus (cls), collateral eminence (cle), and hippocampal formation (hpf) is shown. The choroidal fissure (chf) is a constant anatomical landmark on the medial and upper most parts of the mesial temporal lobe, in close relationship with the ambient cistern (ac). ESIA should be considered for lesions medial to the LOTS C) Superior view of a dry skull base highlighting the middle fossa and its close relationship with the tentorial surface of the cerebellum (tsc). The main landmarks of the epidural sub temporal approach are marked: foramen ovale (fO), petrous bone body (pB) and petrous ridge (pr), where the superior petrosal sinus lies.

The middle cranial fossa floor is widest anteriorly and becomes progressively narrower posteriorly. The opposite is true for the tentorium. It is widest posteriorly, passing across to the contralateral side behind the brainstem, but narrows anteriorly as it stretches lateral the midbrain and attaches to the anterior clinoid process. Thus, the more posterior the target lesion, the more the inter-tentorial plane must be developed to reach it. The SPS marks the level where the temporal dura transitions into the TTL and where the presigmoid dura transitions into the PFTL. It is oriented obliquely along the petrous ridge and is the lateral attachment of the tentorium to the petrous bone, making it a good approximation for a line that separates the anterolateral and posteromedial halves of the basal temporal lobe (Fig. 4). The inferior temporal gyrus, fusiform gyrus, PG, lateral occipito-temporal sulcus, and collateral sulcus are all oriented in an anterior-posterior direction. As a result, the SPS is at the level of the inferior temporal gyrus posteriorly near the transverse sinus, but it is at the level of the uncus anteriorly near its origin. The oblique orientation of the petrous bone, SPS, and lateral tentorium compared to the anterior-posterior direction of the PG, fusiform gyrus, collateral sulcus, and lateral occipito-temporal sulcus is a critical anatomical relationship to understand for this approach, especially when determining the proper location for the dural incision (Fig. 4).

## 4 Discussion

Tentorial peeling allows access to the MTL, LM, CC, and/or AC without transgression of normal brain and can be done without any drilling of the petrous temporal bone. Like the classic subtemporal approach, it provides a short working distance with excellent exposure in the anterior-posterior plane. Peeling of the tentorium and approaching this region epidurally protects the temporal lobe and vein of Labbé. As the vein of Labbé and basal temporal veins remained covered by dura, they are not stretched, and their drainage is left undisturbed. Furthermore, by preserving a protective layer of dura around the temporal lobe, risk of direct retraction and heat injury is significantly reduced compared to the classic intradural subtemporal approach.

### 4.1 Sulcogyral and white matter anatomy

**Fig. 5:**
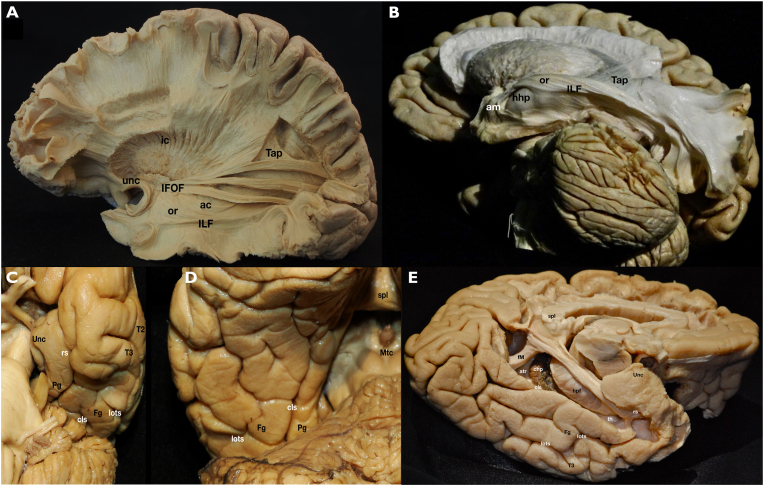
Anatomical white matter specimens. (A) Lateral aspect of a left cerebral hemisphere after removing the most lateral tracts. The white matter fasciculi covering the temporal horn and atrium of the temporal lobe are exposed. Some of them are part of the ventral stream: inferior frontooccipital (IFOF) and inferior longitudinal (ILF) fasciculi. Their close relationship with the internal capsule (ic), uncinate fascicle (unc), anterior commissure (ac), optic radiations (or) and tapetum (Tap) are demonstrated. (B) A more basal view of the same structures is exposed in this dissection. A direct access to the amygdala (am) and head of hippocampus (hhp) is shown avoiding crossing the previously described lateral fibers. C) Anterobasal view of the left temporal lobe anterior half. The uncus (Unc) of the temporal lobe as well as the anterior third of the parahipopcampal gyrus (Pg) are seen, separated from the fusiform gyrus (Fg) anteriorly by the rhinal sulcus (rs) and posteriorly by the collateral sulcus (cls). The lateral occipitotemporal sulcus (lots) divides the fusiform and inferior temporal gyri. D) Posterobasal view of the left temporal lobe posterior half. The posterior extensions of the parahippocampal and fusiform gyri as well as the lateral occipitotemporal and collateral sulci are seen. The relationship of these structures with the splenium (spl) and midbrain tectum (Mtc) are shown. E) A mediobasal view of the left hemisphere is demonstrated. The floor of the temporal horn (th) and atrium (atr) is exposed after removing the parahippocampal gyrus. The hippocampal formation (hpf), choroid plexus (chp) and forceps major (fM) of the corpus callosum are shown.

**Fig. 6:**
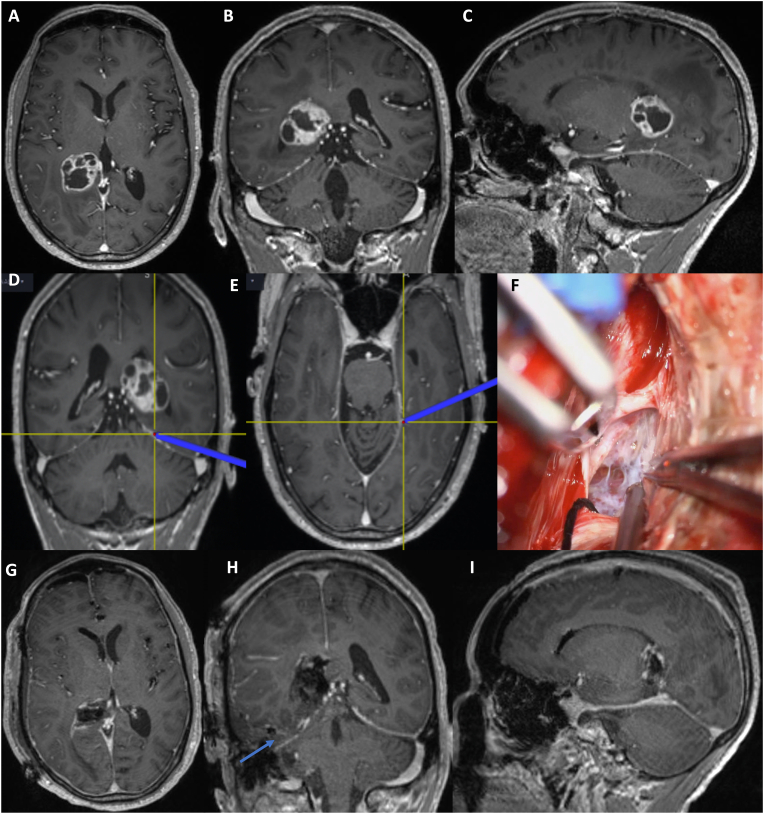
Clinical case illustration: A 70-year-old male presented with 6 months history of headaches, intermittent vertigo, and mild cognitive decline. Imaging revealed a contrast enhancing heterogenous lesion of the parahippocampal gyrus extending to the posterior part of the temporal horn and atrium on the right side (A, B, C). An epidural subtemporal intertentorial approach (ESIA) was performed. Images D and E reveal the medial extent of the intertentorial dissection, shown by the intraoperative navigation pointer. The dura was opened with a linear incision in the temporal tentorial leaf (TTL) at this location. Opening of the TTL revealed the ambient cistern (F). Post-op axial, coronal, and sagittal T1 MRI with Gadolinium contrast showing near total resection of the tumor are seen in images G, H, I. Blue arrow indicates the inter-tentorial space utilized to access the tumor (H). Pathology revealed IDH wild type, non-methylated MGMT, WHO Grade IV glioblastoma.

The sulcogyral anatomy of the basal lobe from lateral to medial includes the inferior temporal gyrus (T3), the lateral occipital temporal sulcus (LOTS), the fusiform gyrus (FG), the rhinal sulcus (RS) anteriorly and collateral sulcus (CS) posteriorly, and the parahippocampal gyrus (PG) (Fig. 4, Fig. 5). The ESIA can approach MTL lesions through the LOTS, FG, RS, CS, or PG, preserving lateral temporal cortex, vasculature, and white matter tracts. For lesions lateral to the LOTS, the benefit of the ESIA is lower, as there is less normal brain that must be crossed and preserved to reach the lesion. Several important white matter tracts pass through or near the superior and lateral temporal lobe (Fig. 5). The optic radiations originate in the lateral geniculate body and pass through the superior and superolateral temporal lobe to reach the occipital cortex. The inferior longitudinal fasciculus (ILF) is a bidirectional tract in the lateral temporal lobe that connects the posterior temporal lobe and occipital lobe to the anterior temporal lobe. The inferior fronto-occipital fasciculus (IFOF) connects the occipital and parietal lobes to the inferior frontal lobe and runs through the infero-lateral insula just supero-medial to the superior temporal gyrus. By approaching the MTL and LM from the basal surface, these important white matter tracts are avoided and preserved (see Fig. 6).

### 4.2 Potential target lesions

There are several different pathologies for which this approach could be useful. Cavernomas, gliomas, or other tumors of the MTL or LM could be directly accessed via the shortest possible working distance without any exposure of the fusiform gyrus or vein of Labbé. P2 aneurysms could potentially be treated with this approach, as the P2 segment of the PCA is well exposed in the crural and ambient cisterns, and proximal control is readily accessible. Selective amygdalohippocampectomy for mesial temporal lobe epilepsy is an interesting potential target, either through a trans-collateral sulcus and/or trans-PG route. The superior to inferior angle required to enter the temporal horn through the collateral sulcus is a difficult angle to obtain with this approach, as this superior trajectory is limited by the floor of the middle fossa and zygoma. Removal of the zygoma could improve this angle. Entry into the temporal horn more laterally through the fusiform gyrus could also improve the operative angle and make amygdalohippocampectomy possible, but the further lateral the entry point, the greater the transgression of normal brain. More anatomic studies and clinical cases are needed to investigate this. Tentorial AV fistulas are a contraindication as these typically have large arterial feeders from the tentorium along with high volume venous tentorial drainage that would render this approach unfavorable.

### 4.3 Surgical considerations

There are several key considerations for optimizing this technique. Given the amount of dural dissection required, it is very important to preserve the dura during craniotomy. A wide dural elevation along the middle fossa floor and a wide tentorial peeling in the anterior-posterior direction allows better mobilization of the temporal lobe, improves visualization, and lessens the amount of epidural retraction. The posterior limit of the tentorial peeling must not disrupt the entry point of the drainage of the basal temporal veins into the transverse sinus. The location and drainage of the vein of Labbé should be carefully studied on pre-op imaging.

The SPS must be clearly identified to guide the proper location of the incision needed to start the tentorial peeling. If the peeling is performed in the wrong plane, the SPS or cavernous sinus can be opened, or the dissection can quickly become intradural. We have found it easiest to identify the SPS and enter the proper plane (superior to the SPS and in between the PFTL and TTL) at the posterior limit of the V3. If there is any difficulty in identifying the superior edge of the SPS, a small amount of the petrous ridge could be drilled to definitively visualize and confirm the position of the SPS prior to starting the tentorial peeling, although this was not necessary in our dissections.

Exposure of V3 is also helpful for verifying the course of the trochlear nerve. In our dissections, an imaginary line that bisects and runs parallel to V3, continued medially to the incisura approximated the location of the trochlear nerve entrance into its groove under the tentorium. An imaginary line parallel to the posterior edge of V3 continued to the incisura was a good estimate for the posterior margin of the incisura, where the tentorium extends across to the contralateral side behind the quadrigeminal cistern. Thus, the peeling can be extended much further here than at the level of V3, and the posterior margin of the incisura should ideally correspond to the midpoint of the peeling in the anterior-posterior plane.

The ESIA may be technically challenging. The epidural dissection required and venous bleeding from the tentorium add complexity. We recommend facial nerve monitoring to help prevent injury to the GSPN and GG during epidural dissection. Careful review of the preoperative imaging is crucial for selecting the appropriate patients for this approach. The slope of the tentorium should be taken into consideration. The steeper the tentorium, the more difficult this approach will be due to the increased difficulty in obtaining a clear line of sight to the pathology (Lafazanos et al., 2015). The slope of the tentorium also increases medially. For an especially steep tentorium, it may be difficult to obtain the proper inferior to superiorly trajectory between the peeled layers of the tentorium that is required for visualization. Partial removal of the zygoma may facilitate a better line of sight in this situation by allowing better visualization superiorly. It is also important to study the location of the MTL and LM relative to the tentorium, to help locate where the site of the incision of the TTL should be to best allow exposure of the target tissue. Stereotactic navigation would be helpful for the medial-lateral and anterior-posterior directions but would be inaccurate in the superior-inferior plane as the tentorium is peeled and the temporal lobe is lifted. In addition, the ultrasound can be utilized to identify the amount of peeling necessary to reach the target lesion. By placing the ultrasound over the extradural temporal lobe, the extent of peeling relative to the intradural structures can be identified in real time, and both the tentorial peeling and the dural opening of the TTL can be optimized.

Venous anatomy must be closely analyzed on pre-operative imaging. Tentorial venous lakes are a common anatomical variant (Shapiro et al., 2020; Muthukumar and Palaniappan, 1998). Typical tentorium venous outflow drains into the superior petrosal and transverse sinus laterally, and the straight sinus medially. The presence of tentorial venous lakes would increase the difficulty of ESIA, but is not necessarily a contraindication, as occlusion of a tentorial lake is usually well tolerated (Muthukumar and Palaniappan, 1998). Tentorial venous bleeding can usually be managed easily with standard hemostatic agents and tamponade. The course of the vein of Labbé is highly variable, with reported patterns including dominant, non-dominant, bilateral, unilateral, and even absent variants (Avci et al., 2011). In some patients, the vein of Labbé or vein of Rosenthal can drain into the SPS (Muthukumar and Palaniappan, 1998; Gutierrez et al., 2020; Sakata et al., 2000). In these cases, care needs to be taken to preserve the SPS.

### 4.4 Comparison to alternative surgical approaches

Many surgical approaches to the MTL and LM are used in neurosurgical practice (Schmeiser et al., 2017; Campero et al., 2006). There are five main categories of approaches for accessing this region (Table 1). The most common approach is a transcortical approach through the inferior temporal gyrus, middle temporal gyrus, or superior temporal sulcus, which puts the lateral neocortex and white matter tracts at risk (Olivier, 2000; Wheatley, 2008) (Fig. 5). The trans-sylvian approach splits the Sylvian fissure widely, putting vascular structures at risk (Wieser and Yaşargil, 1982; Yaşargil et al., 1993). The classic subtemporal approach risks injury to the basal temporal lobe neocortex and temporal veins (Hori et al., 1993). The transorbital approach has a narrow surgical corridor, limited exposure, and cannot access the posterior part of the medial temporal lobe (Monsour et al., 2025). The only direct approach to the MTL and LM comparable to the ESIA is the supracerebellar transtentorial approach. However, it risks injury to the cerebellum, cranial nerves and posterior fossa veins and cannot effectively reach the anterior parts of the MTL or LM (de Oliveira et al., 2012; Giammattei et al., 2021; Gonzalez-Lopez et al., 2021; Yonekawa et al., 2001). Advances in use of the endoscope to assist with this approach may mitigate these risks (Coca et al., 2022). The ESIA therefore, has the potential to directly access the MTL and LM while minimizing the risk of damage to the temporal neocortex, cerebellum, white matter tracts, and neurovascular structures.

**Table 1:** Surgical approaches to the mesiotemporal lobe and the lateral midbrain.

Approach	Advantages	Disadvantages
ESIA	Direct and short trajectory, Cortex and veins protected by dura	Requires skull base techniques and adequate dura, Limited superior reach
Transcortical	Direct and short trajectory, Familiar anatomy, Widest exposure	Cortex and white matter tracts at risk
Subtemporal	Direct and short trajectory, Preserves lateral cortex	Retraction of temporal lobe, vein of Labbé and basal temporal veins at risk, Limited superior reach
Transsylvian	Preserves cortex, Natural corridor, Identifies major vascular structures early	Limited posterior reach, Risk of vasospasm/vascular injury
Transorbital	Small incision, Preserves lateral cortex, Direct anterior/posterior trajectory	Narrow exposure, Limited posterior reach
Supracerebellar Transtentorial	Preserves cortex, Good posterior access, Natural corridor	Long trajectory, Limited anterior reach, Risk to posterior fossa veins

A limitation of this study is the small number of cadaveric specimens (three heads) and only one clinical case. According to the IDEAL framework for surgical innovation, this work corresponds to Stage 1 (Idea), providing an initial description and feasibility assessment of the approach, with further evaluation warranted in larger prospective studies (Dimick et al., 2019).

## 5 Conclusion

The ESIA is a novel approach that has the potential to provide a direct access to lesions of the mesiotemporal lobe, lateral midbrain, crural cistern, and ambient cistern. It enables a short working distance with reduced risk of iatrogenic injury to the temporal lobe and basal temporal veins. Further cadaveric studies and surgical series need to be performed to demonstrate its superiority over the hitherto well described surgical alternatives.

## Declaration of competing interest

The authors declare that they have no known competing financial interests or personal relationships that could have appeared to influence the work reported in this paper.
